# Onion Shipment Volume and Colorectal Cancer Mortality in Japan: An Ecological Study Using a Nationwide Health Insurance Claims Database

**DOI:** 10.7759/cureus.92189

**Published:** 2025-09-12

**Authors:** Ryosuke Shinkai, Takashi Tomita

**Affiliations:** 1 Pharmacology, International University of Health and Welfare, Narita, JPN; 2 Pharmacy, Sanno Hospital, Minato, JPN

**Keywords:** allicin, colorectal cancer, ndb open data japan, onion, shipment stability

## Abstract

Aim: Colorectal cancer is a leading cause of cancer-related mortality in Japan, particularly among older adults. While vegetable intake has been implicated in cancer prevention, most previous studies relied on self-reported dietary surveys, which are prone to recall bias. We aimed to examine the association between onion shipment volume, a novel proxy for consumption opportunity, and age-standardized colorectal cancer mortality among elderly populations in Japan.

Materials and methods: This nationwide ecological cross-sectional study analyzed all 47 prefectures of Japan. Shipment data for onions and other sulfur-containing vegetables (garlic, Chinese chives, green onions) in 2022 were obtained from the Ministry of Agriculture, Forestry and Fisheries. Age-standardized colorectal cancer mortality among individuals aged ≥75 years was obtained from the National Cancer Center. Data were evaluated using visual inspection, box plot analysis, Kruskal-Wallis tests, effect size (Cohen's d), and Spearman's correlation coefficients.

Results: Prefectures with higher onion shipment volumes tended to have lower colorectal cancer mortality rates. Although the difference across prefectures was not statistically significant (Kruskal-Wallis p = 0.643), the effect size was moderate (Cohen's d = 0.524). Spearman's correlation indicated a weak negative association between onion shipments and mortality (r = -0.205, p = 0.168). No consistent associations were observed for other sulfur-containing vegetables.

Conclusion: By using objective shipment data instead of self-reported dietary surveys, this study provides a novel methodological approach to ecological dietary assessment. The findings suggest a possible inverse relationship between onion consumption and colorectal cancer mortality in older adults. Future individual-level studies are warranted to validate these ecological trends and investigate underlying biological mechanisms.

## Introduction

Colorectal cancer has one of the highest incidence and mortality rates in Japan, particularly among older adults, making it a serious public health concern. Dietary improvement is considered an effective strategy for preventing colorectal cancer [1,2], with increasing attention being paid to the association between vegetable intake and cancer risk [2].

Ecological studies, while valuable for identifying population-level associations, inherently carry limitations, such as the ecological fallacy, which precludes direct inference at the individual level. Thus, findings should be interpreted with caution and considered hypothesis-generating rather than confirmatory.

Sulfur-containing compounds found in vegetables such as garlic and onions (e.g., allicin) have demonstrable antioxidant [3] and anti-tumor properties [4], suggesting their potential role in cancer prevention. Onions, in particular, have shown anticarcinogenic effects in both in vitro [5] and in vivo studies [6], and some observational studies involving human populations have also reported beneficial associations [7-10]. Allicin may reduce oxidative stress and prevent DNA damage [4] and has been shown to induce apoptosis in colorectal cancer cells [3]. Furthermore, in vivo experiments in mouse models have demonstrated suppression of tumor formation following onion extract administration [6].

However, despite growing interest in sulfur-rich foods, few epidemiological studies in Japan have evaluated regional variations in onion consumption and their relationship with health outcomes [11]. Most existing research relies on dietary questionnaires [12], which may be subject to recall bias and limited comparability across regions [13].

In this nationwide ecological cross-sectional study of all 47 prefectures in Japan, we aimed to specifically examine the association between onion shipments, used as a proxy indicator of regional onion consumption, and age-standardized colorectal cancer mortality rates among individuals aged ≥75 years. To our knowledge, this is the first study in Japan to evaluate this relationship using actual shipment-based regional data, thereby providing a novel population-level perspective. All analyses and figure creations were conducted using EZR (version 1.61; Saitama Medical Center, Jichi Medical University) [14] and QGIS (version 3.28; Open Source Geospatial Foundation) [15].

Although some observational studies have indicated that allium vegetable consumption may reduce the risk of colorectal cancer [7,9], the findings remain inconsistent [16-18]. Moreover, detailed analyses of regional disparities in Japan are limited. This study provides novel data to address this gap by combining objective, regionally stratified shipment data with standardized mortality statistics.

## Materials and methods

Prefectural-level shipment data for 2022 were obtained from the "Crop Statistics Survey," published by the Ministry of Agriculture, Forestry and Fisheries of Japan [19]. Shipment volumes (in tons) pertaining to onions, garlic, chives, and leeks were collected. Age-standardized mortality rates for colorectal cancer in individuals aged ≥75 years were retrieved from the National Cancer Center Japan (NCCJ) Cancer Information Service [20].

Both onion shipment volumes and colorectal cancer mortality rates were divided into ten categories to create choropleth maps for visual comparison. Onion shipments were further classified into quintiles, and the distribution of colorectal cancer mortality was compared across the groups using box plots. Intergroup differences were evaluated using the Kruskal-Wallis test, and associations were assessed using Spearman's rank correlation coefficient. Effect size (Cohen's d) was calculated to compare the lowest and highest quintiles. All analyses and figure creations were conducted using EZR [14] and QGIS [15].

This study used only publicly available aggregated data containing no personally identifiable information. Therefore, in accordance with the national guidelines, formal ethical reviews and informed consent were not required.

## Results

**Figure 1:**
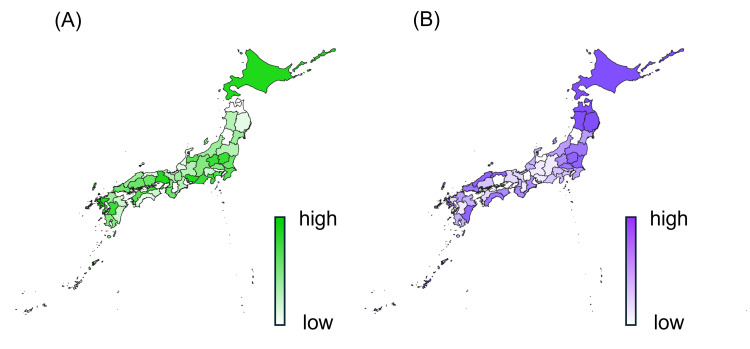
Geographic distributions of onion shipments and colorectal cancer mortality rates among older adults in Japan. (A) Onion shipment volume per 100,000 population across all prefectures in Japan. (B) Age-standardized colorectal cancer mortality rate per 100,000 population among individuals aged 75 years or older in 2022. Both are visualized using gradient choropleth maps.

**Figure 2:**
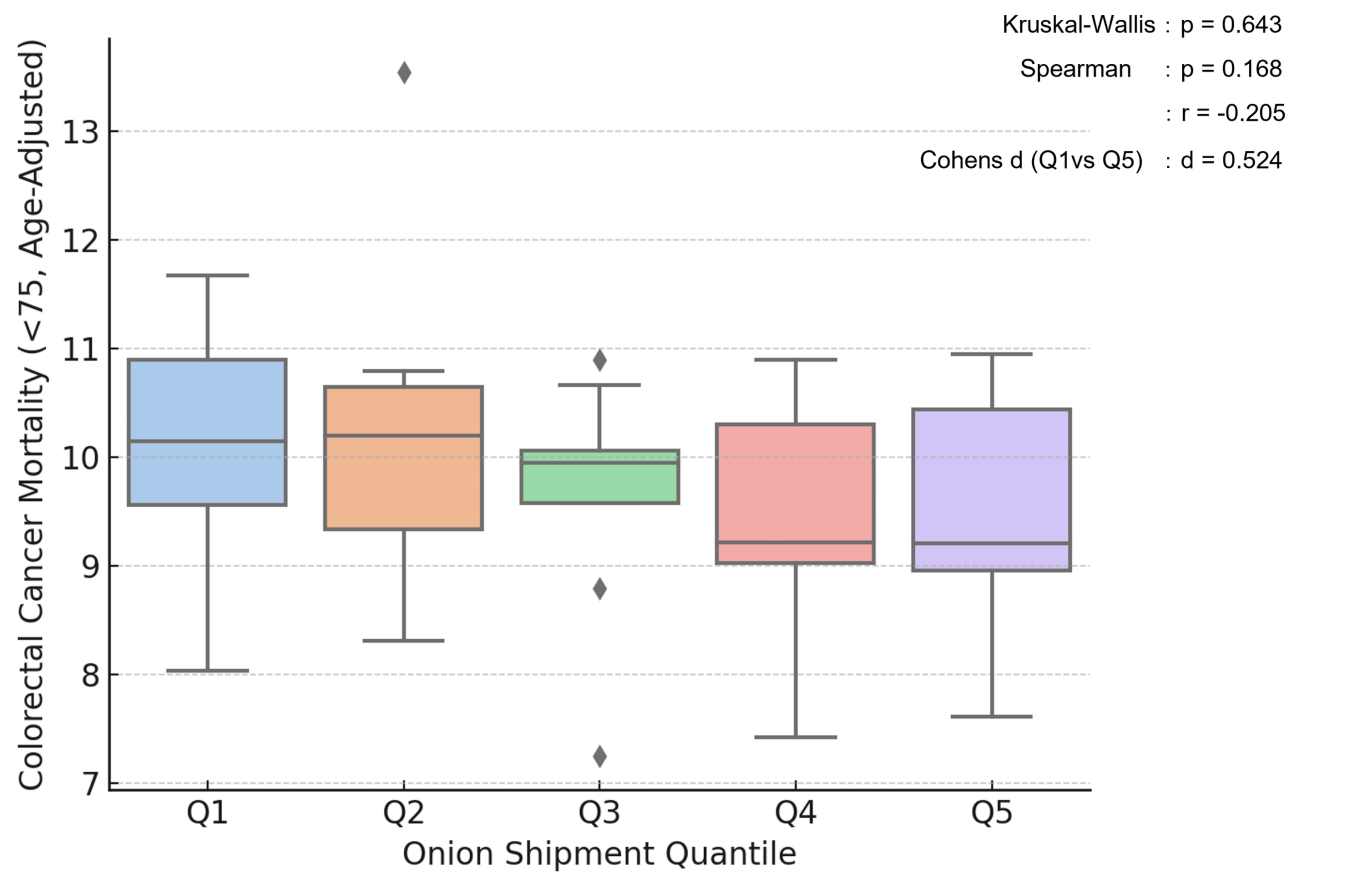
Comparison of colorectal cancer mortality by onion shipment quintiles. Box plot of age-standardized colorectal cancer mortality by quintiles (Q1–Q5) of onion shipment volume. Kruskal-Wallis test: p = 0.643. Cohen's d (Q1 vs Q5): d = 0.524. Spearman's rank correlation coefficient: r = –0.205 (p = 0.168).

Visual inspection of onion shipment (Figure 1A) and colorectal cancer mortality rates (Figure 1B) revealed that regions with higher shipment volumes tended to have lower mortality rates. Box plot analysis (Figure 2) showed that the highest quintile of onion shipment had a lower median mortality rate than the lowest quintile. Although this difference was not statistically significant (Kruskal-Wallis test, p = 0.643), the Spearman correlation coefficient was r = −0.205 (p = 0.168), suggesting a trend toward an inverse relationship. The effect size (Cohen's d) between the highest and lowest quintiles was 0.524, indicating a moderate effect.

**Figure 3:**
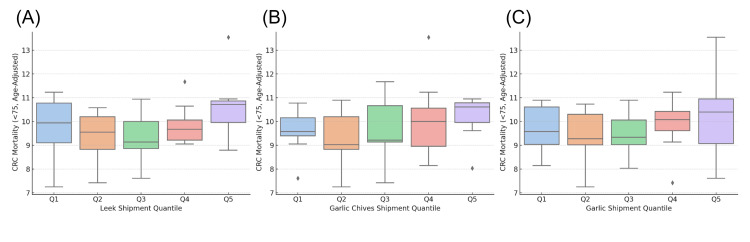
Association between allium vegetable shipment volumes and age-standardized colorectal cancer mortality in Japan. (A) Box plot of age-standardized colorectal cancer mortality by quintiles (Q1–Q5) of garlic shipment volume. (B) Box plot by quintiles of Chinese chive shipment volume. (C) Box plot by quintiles of green onion shipment volume. All figures are based on prefectural data and visually depict differences in mortality according to stratified shipment levels.

In additional analyses, no clear trends were observed between the mortality rate and the shipment volumes of other sulfur-containing vegetables (Figure 3).

## Discussion

This study utilized publicly available data to perform a comprehensive ecological evaluation across Japan, visualizing regional disparities that are difficult to capture in individual-level studies. Unlike traditional dietary surveys using questionnaires, we used objective shipment data as a proxy for consumption, enabling a more consistent assessment across regions. The study's strengths also include the comparison with other sulfur-containing vegetables and the use of multiple statistical approaches, including effect size and correlation coefficients, to provide scientific interpretation even in the absence of statistical significance.

Although no statistically significant differences were observed, exploratory analyses indicated a negative trend (Spearman r = −0.205, p = 0.168) and a moderate effect size (Cohen's d = 0.524) between the highest and lowest quintiles of onion shipment. These results should be interpreted with caution, as they do not demonstrate a definitive association but may warrant further investigation. Allicin and other sulfur compounds in onions have been reported to exert antioxidant [3] and anti-tumor properties [4]. Allicin may reduce oxidative stress and prevent DNA damage [3,4], and has been shown to induce apoptosis in colorectal cancer cells in vitro [5]. Furthermore, in vivo studies in mouse models have demonstrated suppression of tumor formation following onion extract administration [6]. These biological mechanisms may be consistent with the regional trends observed in this study [21,22].

While some observational studies have indicated that Allium vegetable consumption may reduce colorectal cancer risk, the findings remain inconsistent [7,10,16]. Detailed analyses of regional disparities in Japan have been limited, and this study contributes novel data addressing that gap.

This study has several limitations. First, as an ecological study, it does not account for individual-level factors such as dietary intake or lifestyle, raising the possibility of ecological fallacy [23]. Second, confounding factors that influence colorectal cancer mortality, such as smoking, alcohol use, screening uptake, and access to medical care, could not be fully adjusted for. Third, shipment volumes do not directly equate to local consumption, as onions may be shipped outside their production region. However, shipment data are still considered a reasonable proxy for local dietary tendencies, especially since we observed consistent trends between shipment and mortality.

Additionally, allicin is known to degrade easily with heat, and its bioactivity may be lost during cooking or processing [24]. This discrepancy between shipment volume and actual intake of bioactive compounds may partly explain why no clear associations were found with other sulfur-containing vegetables.

Future research should aim to validate these findings through individual-level epidemiological or interventional studies, as well as molecular epidemiology investigating associations between sulfur-containing compounds and cancer-related biomarkers. In particular, future studies employing validated food frequency questionnaires or 24-hour dietary recalls could complement ecological analyses and help confirm the associations suggested by this study.

## Conclusions

This nationwide ecological study demonstrated the feasibility of using objective annual onion shipment data as a novel and quantifiable proxy for regional consumption to assess age-standardized colorectal cancer mortality in individuals aged ≥75 years. By avoiding the limitations of self-reported dietary questionnaires, this approach enabled consistent regional comparisons and revealed a moderate, albeit non-significant, inverse association between onion shipment volume and mortality. These findings contribute a new methodological perspective to dietary epidemiology and suggest that increased onion consumption may be linked to reduced colorectal cancer mortality in older adults. Further individual-level and mechanistic studies are warranted to validate these ecological trends and clarify the underlying biological pathways.
